# Psychometric properties of Persian version of advance care planning questionnaire among older adults in Iran

**DOI:** 10.1186/s12877-024-04976-5

**Published:** 2024-05-06

**Authors:** Mobina Golmohammadi, Salman Barasteh, Mohsen Mollahadi, Shadi Baba Ali, Abbas Ebadi

**Affiliations:** 1 Student Research Committee, Baqiyatallah University of Medical Sciences, Tehran, Iran; 2 Health Management Research Center, Baqiyatallah University of Medical Sciences, Tehran, Iran; 3 Nursing Faculty, Baqiyatallah University of Medical Sciences, Tehran, Iran; 4 Exercise Physiology Research Center, Life Style Institute, Nursing Faculty, Baqiyatallah University of Medical Sciences, Tehran, Iran; 5 Department of Internal Medicine, Imam Khomeini Hospital, School of Medicine, Tehran University of Medical Sciences, Tehran, Iran; 6 Behavioral Sciences Research Center, Life Style Institute, Baqiyatallah University of Medical Sciences, Tehran, Iran

**Keywords:** Aging, Advanced care planning, End-of-life care, Psychometrics, Palliative care, Hospice, Advance directives

## Abstract

**Introduction:**

Advanced age is associated with life-threatening conditions at the end of life. Many of these persons at the end of their lives cannot make decisions because of the variable consciousness. They are able to make decisions and identify their care priorities, in a process called advanced care planning. So, an instrument is required for investigating ACP of the elderly population. This study was performed to determine the psychometric properties of the Persian version of the advanced care planning questionnaire(ACPQ) in elderly population referring to Tehran.

**Method:**

This methodological study was performed in five hospitals in 2021–2022. A total of 390 eligible elderlies were included. The psychometric assessment including translation, face validity, content validity were performed Alsothe exploratory factor analysis and confirmatory factor analysis were assessed. Reliability were done by internal consistency by assessing Cronbach alpha and stability was performed using test-retest.

**Results:**

The face validity of the instrument was performed with minor changes. The content validity index for all of the items was above 0.79. In EFA four factors was extracted also CFA showed that the four-factor model has a good fit of the data (RMSEA: 0.04; NFI: 0.97 CFI: 0.99; IFI: 0.99; RFI: 0.96; AGFI: 0.87; GFI 0/90; standardized RMR: 0.02). Cronbach alpha and ICC were 0.72–0.94 and 0.85–0.96, respectively.

**Conclusion:**

The Persian version of the advance care planning questionnaire has desirable psychometric properties for measuring the advanced care planning of the elderly population. In addition, healthcare providers in Iran can employ this questionnaire in their practice and research.

## Introduction

Advanced age phenomenon is the result of the natural course of time, leading to physiological, psychological, and social changes in the elderly population [1]. According to the WHO regarding the elderly’s population by 2030 out of every six persons in the world one will be 60 years old or older [2]. In Iran, according to a census performed in 2018, 10% of the entire population of the country is elderly, and it is predicted that by 2041, it would reach 20% and by 2050 reach 31.2% [3].

With aging, the probability of developing chronic diseases increases significantly among the elderly population [4, 5]. Suffering from diseases would confront the elderly person to limitations at the end of life [6]. Studies show that the end-of-life care needs of the elderly patients do not receive the attention they deserve [7, 8]. It is estimated that close to 30 million people worldwide have an immediate need to end-of-life care, with 69% of them being 60 years above [9].

In Iran, attention to end-of-life issues and problems can be enhanced through an approach called palliative care [10]. The study by Fereidouni et al. in Iran indicated that in order to support patients, care providers and families of patients, the place where palliative care is provided for patients at the end-of-life should be compatible with their wishes and preferences [11]. Furthermore, elderly patients at the end-of-life should share their preferences with others around them, or choose a person as a proxy decision-maker [12]. Accordingly, studies suggest that the elderlies who can make decisions should plan for their preferences for end-of-life care [10, 13].

These measures are generally known as advanced care planning (ACP), with the aim of helping patients who lack decision-making capacity at the end-of-life about future care [14] and it is considered as an essential step for achieving “a good death” [15, 16]. ACP is defined as the process of making end-of-life treatment preferences, communicating goals of care, ordering life-sustaining treatments, and completing advance directives (ADs) [13].

In Iran, like other Muslim countries, palliative care planning faces various challenges. For some Muslims, ACP is a vague concept and rarely discussed [17]. In one study found that Muslim people put off uncomfortable conversations about care planning until they become ill or suddenly become unable to express their wishes [18]. Despite the interesting of Muslim community in learning and receiving the concept of ACP [19], ACP in Iran, does not exist in a coherent way, but researchers and clinician have separately and limitedly investigated the dimensions of it, including palliative care [10, 20] and hospice care [15, 21], preferred place of care and preferred place of death [11], have a kind of ADs as Vasiyyah [22], and do not resuscitation [23] in their practice and research [24].

Considering that achieving ACP in the elderly requires older people’s awareness and acceptance evaluation, so far, numerous instruments have been developed for evaluating views as well as advanced care planning. The most important instruments include the Cultural values and beliefs scale designed in the United States [25], The ethnicity and attitudes toward advance care directives questionnaires designed in the South Korea [26], Asian American quality of life survey designed in China [27], and Advance Care Planning Questionnaire (ACPQ) designed in Malaysia [28]. ACPQ is one of the instruments for examining the awareness and acceptance of the patient for receiving end-of-life care. This instrument was designed by Lai et al. in Malaysia in 2016. The aim of this questionnaire was to assess awareness and acceptance of the elderly about advanced care planning [28]. This instrument has been developed in Malaysia as a Muslim country, which is a shared feature with Iran. This, in turn, makes this instrument a means for better achieving its items [29].

So far, in Iran attention to patients’ preferences for end-of-life care is in its early stages and extensive search across the literature suggests that the studies performed in the country on the knowledge and attitude to ACP at end of life are very limited and there is no standard instrument about its measurement in Iran. Thus, the present study was done to examine the psychometric properties of Persian version of ACPQ in the elderly referring to hospitals in Tehran.

## Method

### Study design

This methodological study has dealt with translation and investigation of the psychometric properties of ACPQ in Persian in 2021–2022.

### Study population/sampling

The research population consisted on 390 elderlies referring to the hospitalization wards and outpatient clinics of five hospitals in Tehran including Baghiatallah, Fajr, Shohadaye Tajrish, Imam Khomeini, and Firoozgar hospitals. The sampling was done in 2021–2022 using convenience sampling method. Sampling in psychometric studies commonly is convenience and done to assess EFA and CFA base on the number of questionnaire items [30]. The inclusion criteria included age at least 60 years, willingness to participate in the study, minimum literacy of reading and writing, lack of psychological disorders according to the medical file or patient’s self-expression, ability to speak Persian, and suitable physical conditions of the patient to respond to the questions. The exclusion criteria included lack of willingness to continue participation and filling in the questionnaire incompletely.

### Study instrument

#### Demographic information questionnaire

The demographic information in this study included age, gender, marital status, and level of education of the eligible elderlies to be included in the study.

#### Advance care planning questionnaire

ACPQ was designed by Lai et al. in Malaysia to evaluate the knowledge and attitude of the elderly to advanced care planning. This questionnaire includes 16 items in four dimensions of “feelings regarding advance care planning” (5 items), “justifications for advance care planning” (4 items), and “justifications for not having advance care planning” (7 items). The questionnaire items are score as five-point Likert scale as follows: 1 = strongly agree, 2 = Agree, 3 = Do not know, 4 = Disagree, 5 = strongly disagree.

All participants were supposed to respond to the five items of “feelings regarding advance care planning” domain. Those who choose strongly agree and agree options should only answer four items of justifications for advance care planning, and those who answer the other options of the Likert scale should only answer 7 items of justifications for not having advance care planning [28].

### Translation procedure

The translation process was done as forward-backward. In the forward translation stage, the original English version, after acquiring permission from its developer, was translated into Persian according to the International Quality of Life Assessment (IQOLA) separately by two translators were proficient in English [31]. To acquiring permission, we sent an email to Pauline, the developer of the original version of ACPQ, and after signing the agreement, the permission was granted. Next, in the incorporation stage, in a meeting in the presence of the researchers, the two translated versions were investigated and ultimately with the agreement of the researchers, a preliminary common translation was obtained. In the backward translation stage, the common Persian translation prepared in the previous stage was re-translated back to English by two natives proficient in Persian and English who were not aware of the original version, whereby an English version was achieved. For comparison, the two English translated versions obtained in the previous stage were sent to the developer of the scale. The sent questionnaire was investigated against the original version of the questionnaire by the developer conceptually and confirmed further. Thereafter, the stages of cultural adaptation and other psychometric properties were performed as follows.

### Face and content validity

After completion of the translation procedure, to explore the quantitative face validity, cognitive interviews were used. Cognitive interview was performed to identify the source of error in the questionnaire with a focus on the cognitive process of the respondents during completion of the questionnaire [32]. Accordingly, face-to-face interview was performed with 10 elderlies who were different regarding level of education as well as socio-economic status. They were asked to evaluate the legibility, clarity, and structure of items, as well as convenience of comprehension, difficulty of items, confusing words, classification of items, ease of responding, linguistic forms, and wording [33].

To explore the content validity, the Persian version of the advance care planning questionnaire was given to 10 experts in palliative care. They were asked to investigate the relevancy of the items using a 4-point Likert scale ranging from irrelevant = 1, somehow relevant = 2, is relevant = 3, absolutely relevant = 4. Ultimately, CVI score was calculated for the items. CVI above 0.79 is suitable, 0.7–0.79 requires a revision and correction, while scores lower than 0.7 are unacceptable and should be eliminated [34, 35].

### Item analysis

The aim of item analysis at this stage is to create a specific set of items for each dimension of the construct. Correlation degree lower than 0.3 indicates limited correlation with the construct [34]. In this section, item analysis was performed with 30 elderlies participating in the study using the loop method and with analysis of items in SPSS software, with Cronbach alpha also reported. Correlation of all items was obtained above 0.3.

### Construct validity

To explore the construct validity of this scale, exploratory factor analysis (EFA) and confirmatory factor analysis (CFA) were assessed.

### Exploratory factor analysis (EFA)

Exploratory factor analysis (EFA) is employed for discovering the underlying structure of a relatively large set of variables. The minimum sample size required for EFA is 3–10 participants per each item [36]. To examine the EFA, 230 elderly’s person were included in this study. To explore the adequacy of sampling and suitability of the subjects, Bartlett’s test and Keiser-Meyer-Olkin (KMO) test were done. KMO closer to one is more suitable for performing factor analysis; however, generally a score larger than 0.5 is acceptable and scores greater than 0.7 are more suitable. The Bartlett’s test with significance level below 0.05 is acceptable [37, 38]. The suitable results of the two KMO and Bartlett’s tests indicate existence of desirable correlation matrix for conducting factor analysis [39]. Factor load value refers to the relationship between each factor and each item of the questionnaire. In order for each item to remain in the analysis, that relationship should be suitable. The minimal factor load value in this study was considered 0.3. In case factor load lower than 0.3, the relationship between the item and factor is weak [40, 41]. For factor extraction, considering skewness (± 3) and kurtosis (± 7) indices, maximum likelihood (ML) method was used, while for interpretability of the factors, varimax rotation was employed [42]. For the first time, Thompson propounded orthogonal varimax rotation which is considered the most common technique used in factor analysis which generates independent factorial structures [43].

### Confirmatory factor analysis (CFA)

Following EFA, the factors extracted were evaluated by CFA. For this purpose, 160 other elderlies were included in the study for CFA assessment. CFA was done using least partial squares for assessing the goodness of fit of the proposed model with the data. Accordingly, the significance and intensity of correlation are determined. The fitness indices of the model in CFA are classified into three general categories: (1) absolute fit: root mean square error of approximation (RMSEA), standardized residual squared mean root (SRMR), fitness index (GFI, Chi square), (2) comparative fitness: comparative fitness index (CFI, incremental fitness index (IFI), RFI, normal fitness index (NFI), TLI, and (3) sole fitness: AIC, AGFI, and PNFI [44].

### Reliability

To determine the reliability of ACPQ, internal consistency and stability determination methods were used. For measuring the internal consistency, Cronbach’s alpha coefficient was calculated. To have internal consistency of good and sufficient level, Cronbach alpha coefficient should be between 0.7 and 0.8 [24]. In order to determine the stability of the instrument, test-retest method was used with a sample size of 30 subjects. In this study, the time interval for the retest was considered 14 days, and the scores acquired in these two stages were compared with each other using intra-cluster correlation index (ICC). ICC above 0.8 is considered as desirable [24]. In this research, the total-item correlation was also investigated. The correlation of each item with the total score of the scale was calculated and then based on these correlations; decisions were made on elimination or retainment of the items. All of the items were retained at this stage [45].

In this study, standard error of measurement was also calculated. Small values of this scale for any instrument are important since changes above that are clinically significant. The standard error of measurement is employed for quantification of the accuracy of the score of each subject. To calculate this index, the following formula can be used. In this formula, SD represents standard deviation of the sum of the test and retest subjects [46].

### Ethical considerations

The permission for conductance of the study was taken from ethics committee of Baghiatallah University of medical sciences with the ethics code of IR.BMSU.REC.1400.038. After acquiring written permission through email, from the instrument’s developer, the translation procedure was initiated. For observing ethical principles, the elderlies were included in the study voluntarily. It was also emphasized that lack of participation in the study would have absolutely no effect on their course of care and treatment. In addition, informed consent was taken from all elderlies. Confidentiality of all data and observation of integrity of the utilized data sources were also observed.

### Data analysis

For data analysis, SPSS 26 was used along with the relevant tests together with LISREL 8.8 software. Significance level was considered 0.05.

## Results

### Socio demographic and clinical status

Out of 390 participants in the study, 230 took part in exploratory factor analysis and the other 160 in confirmatory factor analysis. The elderly persons participating in the exploratory factor analysis included 110 women and 120 men with the mean age of 68.26 ± 7.38 with most of them being married (83%). Also 30.4% In the confirmatory factor analysis, 100 participants were female and 60 were male with the mean age of 64.34 ± 4.36, with most being married (79.4%). Table 1 reports the complete information of the participants.

**Table 1:** Rating of exploratory factor analysis (EFA) and confirmatory factor analysis (CFA) based on demographic characteristics

	Category	EFA N (%)		CFA N (%)
Gender	Male	120(52.2%)		60(37.5%)
	Female	110 (47.8%)		100(62.5%)
Age			Mean(SD)	
		68.26 ± 7.38		64.34 ± 4.36
	60–70	159(69.1%)		148(92.5%)
	71–81	54(23.5%)		12(7.5%)
	> 81	17(7.4%)		0
Education	elementary	70(30.4%)		25(15.6%)
	high school	48(20.9%)		95(59.4%)
	diploma	85(37%)		40(25%)
	Master’s/Ph.D.	27(11.7%)		0
Married	Married	191(83%)		127(79.4%)
	Widow/divorced	39(17%)		33(20.6%)

### Face and content validity

The face validity was examined with 10 eligible elderlies via cognitive interview method. Item 12 was modified due to containing medical terminology for the participants in examining the face validity. This item was as “if you have severe dementia = you have developed Alzheimer’s” which changed into “you have developed forgetfulness”. The content validity was checked based on opinions of ten experts in palliative care. Content validity index (CVI) was calculated for the items. All items had score above 0.79, and no item was eliminated at this stage (Table 2).

### Construct validity

#### Exploratory factor analysis

For “feelings regarding advance care planning factor”, the Kaiser-Meyer-Olkin (KMO) was 0.894 Bartlett’s test were significant (*p* < 0.001, x2 = 1410.78 df = 10 × 2 = 1410.78) and the factor “justifications for having advance care planning” has the KMO 0.77 andBartlett’s test were significant (*p* < 0.00, x2 = 137.82 and dF = 6).For the factor “justifications for not having advance care planning”, the KMO 0.685 and Bartlett’s test were significant (*p* < 0.001, x2 = 136.55, df = 21). For extraction of factors, maximum likelihood method, and for interpretability of the factors, Varimax rotation was used (Table 2). Also the four factor “feelings regarding advance care planning”, “justifications for having advance care planning”, “justifications for not having advance care planning”, and “ justifications for not having advance care planning” have 84.47, 49.28, 21.93, and 19.62, respectively.

**Table 2:** EFA of the Persian version of the ACPQ

Factor	Item	Factor loading	Variances
feelings regarding advance care planning	Q8	0.0.90	84.47%
	Q9	0.0.92	
	Q10	0.0.89	
	Q11	0.87	
	Q12	0.88	
justifications for having advance care planning	Q13	0.0.61	49.2849.28%
	Q14	0.68	
	Q15	0.0.58	
	Q16	0.0.49	
justifications for not having advance care planning justifications for not having advance care planning	Q17	0.48	21.93%
	Q18	0.32	
	Q19	0.43	
	Q20	0.40.	
	Q21	0.33	
	Q22	0.39	19.62%
	Q23	0.46	

#### Confirmatory factor analysis (CFA)

**Fig. 1:**
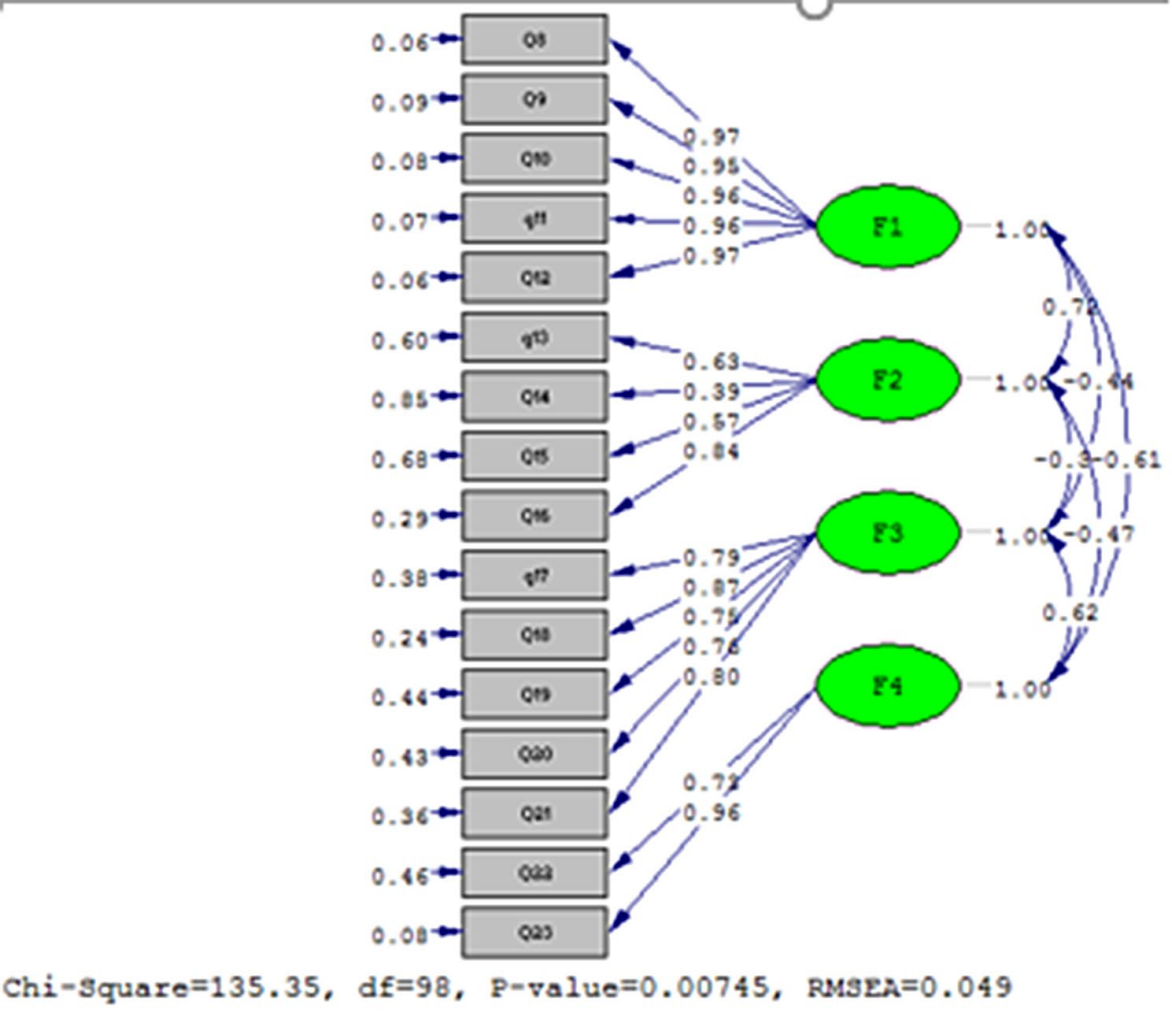
The final structure of the model

Another sample consisting of 160 older adults was selected for CFA. The results of the chi-squared test (x2 = 135.35 and *P* = 0.00) and other fit incises showed that the three-factor model extracted from EFA has a good fit of the data (RMSEA: 0.04; NFI: 0.97 CFI: 0.99; IFI: 0.99; RFI: 0.96; AGFI: 0.87; RMR: 0.077; standardized RMR: 0.02). Finally, the results showed that CFA based on the three-factor model extracted from EFA with the obtained data has a good fit (Fig. 1).

### Reliability

For reliability, internal consistency was obtained with confidence interval 95% (CI) using Cronbach alpha coefficient for factor 1 feelings regarding advance care planning (0.94), factor 2 justifications for advance care planning (0.78), factor 3 with two components justifications for not having advance care planning (fate and religion and avoiding thinking about death) (0.72). Also, ICC for factors 1, 2, and 3 was obtained as 0.96, 0.91, and 0.85, respectively. Other information is presented in Table 3.

**Table 3:** Reliability by the method of internal consistency and relative and absolute stability

SEM^*^	Mean (SD^**^)	CI^***^ (95%)	ICC^****^	Cronbach alpha	items	Original domain
1.02	11.01(5.25)	0.92–0.98	0.96	0.94	8,9,10,11,12	feelings regarding advance care planning
0.84	5.97(2.97)	0.78–0.97	0.91	0.78	13,14,15,16	justifications for advance care planning
1.74	15.16(4.50)	0.50–0.95	0.85	0.72	17,18,19,20,21,22,23	justifications for not having advance care planning

* Standard error of measurement

**standard deviation

***confident interval

****interclass correlation coefficient

## Discussion

The present study was performed to investigate the psychometric properties of the Persian version of ACPQ. According to the results, the face validity was confirmed by the elderly individuals, and the content validity was confirmed by palliative care experts. Furthermore, the results obtained from the construct validity using EFA and CFA indicated that the utilized instrument has a suitable structure. The reliability of the instrument was calculated by internal consistency test (Cronbach alpha coefficient = 0.72–0.94), showing suitable reliability of the instrument. Also, test-retest stability was conducted, which showed the time stability of the instrument.

In the present study, the translation process was performed carefully until achieving a final Persian version. Investigation of the face validity of the instrument using the opinions of 10 eligible elderlies showed that the items were simple and clear, and only in item number 12, some brief changes were made. The content validity was also explored using the opinions of 10 experts in palliative care by calculating CVI. In examination of CVI, all items had a score above 0.79. The face and content validity were examined in the original Malaysian version by two physicians specialized in palliative care, two specialists of geriatric medicine, family medicine specialist, and one family medicine clinical postgraduate candidate. Thereafter, each item was investigated and the relevancy of each item was discussed. Some items were eliminated, some were rewritten, and some new items were added until the panel of experts recognized that ACPQ covers all important areas in ACP [28]. In the study by Lim et al., the translation was performed as back-and-forth, and the English questionnaire was translated to Malaysian. Next, the face and content validity were confirmed by a specialist panel consisting of two first aid physicians, three pharmacists, and one pharmacist currently involved in the field of ACP in Malaysia [47].

In the present study, in EFA, four domains including “feelings regarding advance care planning”, “justifications for advance care planning”, justifications for not having advance care planning: fate and religion”, and “justifications for not having advance care planning: avoid thinking about death” were extracted, which is similar to the original study [28].

Various studies have examined feelings regarding ACP. According to studies, feeling regarding ACP can be unpleasant feeling or positive. In some studies, it has been mentioned that patients feel disturbed [48, 49]. But some studies have also paid attention to the positive side of the feeling, such as the feeling of power [50, 51] Or in Holden Caplan et al.‘s study, most patients felt comfortable talking to their doctor about ACP and EOL decisions and were satisfied with how their doctor talked about EOL. They also felt comfortable discussing their EOL wishes with their trusted physician [52]. In the dimension of “justifications for advance care planning”, there was no change in the Persian version of the tool according to the Malaysian version [53]. According to the results of this study, this dimension was not changed in the Malay version [47].

In the field of justifications for not having advance care planning: fate and religion, studies have mentioned religiosity as an important factor in avoiding ACP. In Martina’s study, patients who believed that life is a sacred loan that must be protected often avoided discussions about limiting invasive interventions and saw the concept of ACP as conflicting with their beliefs [54]. Despite the positive effects of religious beliefs, strong religious beliefs on people’s physical and mental health [55], higher levels of religiosity, reliance on religious coping, conservative faith traditions, and belief in God’s control over lifespan and divine intervention have lower levels of ACP and extreme EOL care preferences [56]. Also, in a study, religious participants indicated that the manner of death and self-medication should be consistent with their religious teachings and values. They often discussed their disease status and treatment plan during hospitalization and preferred comfort care or limited care near EOL because of their faith. However, the results of some studies show that health care providers rarely pay attention to their religious beliefs [57].

In all dimensions, KMO varied between 0.68 and 0.89. This indicates that the size of samples has been sufficient in all dimensions. Bartlett’s test was also significant in all dimensions, suggesting that the correlation between the items has been sufficient for exploratory factor analysis. In line with this study, in the research by Lim et al., again KMO in all dimensions was above 0.6 and the factor loading values were also above 0.4. Furthermore, they reported suitable exploratory factor analysis for the ACPQ [47]. We are not able to compare the study results with more studies since only one study has dealt with translation and psychometric properties determination of this study. Nevertheless, it seems that while extracting four factors similar to the original questionnaire and similar to Lim, the overall framework of the questionnaire has been preserved.

In this study, CFA showed that the three-factor model extracted from EFA has a good fit of the data (RMSEA: 0.04; NFI: 0.97 CFI: 0.99; IFI: 0.99; RFI: 0.96; AGFI: 0.87; GFI 0/90; standardized RMR: 0.02). The four-factor model well confirms the questionnaire. The original study performed in Malaysia had not performed confirmatory validity [28]. However, similarly the study by Lim et al. has also confirmed the four-dimensional model of this instrument [47]. Thus, considering the confirmation of the four dimensions of the model in the present study as well as the original study together with its confirmation in the study by Lim et al., it seems that the four-dimensional model is also suitable in Persian language.

In the present study, reliability measurement of the Persian version was confirmed using internal consistency (Cronbach’s alpha = 0.72–0.94) along with stability ICC = 0.85–0.96, indicating suitable reliability of the instrument. The reliability of the instrument in the Malaysian version was investigated and confirmed using test-retest method with Kohen Kappa coefficient and score (0.738–0.94) as well as Cronbach alpha coefficient (0.637–0.915) [28]. Lim et al. in their study reported that the Cronbach alpha value for four areas ranged from 0.674 and 0.947. In the retest, second-order weighted kappa values for all domains were 0.340–0.674, except for two domains (justifications of not having ACP (fate and religion) and “justifications for not having ACP (avoidance of thinking about death), ranging from 0.20 to 0.46 [47]. Therefore, the instrument enjoys sufficient internal consistency and stability for usage in clinical and research settings.

### Strengths of the study

The low number of items in this instrument has contributed to short completion time, and yet this instrument can also be used for examining the factors associated with advanced care planning. This can be a credible source for investigation of personal preferences in receiving the most suitable treatment and care in terminal stages of life. This questionnaire has not been limited to any special disease or condition or stage of disease. Indeed, it can be employed in other chronic diseases and different stages of the disease as well as healthcare centers including hospitals, geriatric healthcare centers, or hospice.

## Conclusion

ACPQ is a reliable and valid instrument for investigating the awareness and approval of advanced care planning among the elderlies of Iranian society. This can help policymakers to determine the preparation of the elderly for ACP, so that in a not very far future, we can witness development of palliative care and advanced care planning as one of its dimensions. Also, this instrument can be employed in clinical evaluation and for research purposes.

### Limitations

As one of the limitations of this study, it has investigated only the views of the elderly, and conducting some further studies as well as an exploration of the factors associated with advanced care planning according to other patients with different age conditions, or the views of physicians, nurses, and other healthcare providers and its comparison with the present study can be important. Another limitation is that ultimately these results may not be generalizable to all diseases, and it is recommended to conduct similar studies on special chronic or life-threatening diseases. Meanwhile, most respondents consisted of women, which can influence the results. Considering the limitations of access to the elderly eligible to be included in this study at the time of sampling as well as the novel nature of the concept and their familiarization with the concept, the access to the subjects was heavily time-consuming.

## Data Availability

All data generated or analyzed during this study are included in this published article.
